# PtrMYB3, a R2R3-MYB Transcription Factor from *Poncirus trifoliata*, Negatively Regulates Salt Tolerance and Hydrogen Peroxide Scavenging

**DOI:** 10.3390/antiox10091388

**Published:** 2021-08-30

**Authors:** Tonglu Wei, Dalong Guo, Jihong Liu

**Affiliations:** 1 Key Laboratory of Horticultural Plant Biology (MOE), College of Horticulture and Forestry Sciences, Huazhong Agricultural University, Wuhan 430070, China; weitonglu@haust.edu.cn; 2 College of Horticulture and Plant Protection, Henan University of Science and Technology, Luoyang 471023, China; guodalong@haust.edu.cn

**Keywords:** MYB, peroxidase, *Poncirus trifoliata*, salt stress, transcription factor

## Abstract

MYB transcription factors are widely present in plants and play significant roles in abiotic stresses. However, most MYB genes have not been identified in plants and their functions in abiotic stresses are still unknown. In this study, one MYB gene, designated as PtrMYB3, was cloned from trifoliate orange (*Poncirus trifoliata* (L.) Raf.), and its function in salt tolerance was investigated. PtrMYB3 contains a conserved R2R3-MYB domain, which is the typical property of R2R3-MYB subfamily proteins. Expression profiling under abiotic stresses indicated that PtrMYB3 could be induced by salt, dehydration and cold stresses. PtrMYB3 was found to be localized to the nucleus and possessed transactivation activity. Overexpression of PtrMYB3 by genetic transformation in tobacco impaired its salt tolerance, whereas silencing of PtrMYB3 by VIGS (virus-induced gene silencing) in trifoliate orange conferred significantly enhanced salt tolerance, indicating that PtrMYB3 negatively regulates salt tolerance. Furthermore, a peroxidase gene (*PtrPOD*) was found to be greatly upregulated in PtrMYB3-silenced trifoliate orange, and a dual LUC (luciferase) assay confirmed that PtrMYB3 could suppress the expression of *PtrPOD*. The hydrogen peroxide (H_2_O_2_) accumulation in PtrMYB3 transgenic tobacco plants after salt stress was higher than the wild type (WT), further confirming that overexpression of PtrMYB3 inhibited *PtrPOD*-mediated H_2_O_2_ scavenging. Taken together, these results demonstrate that PtrMYB3 negatively regulates salt tolerance, at least in part, due to the excess accumulation of H_2_O_2_.

## 1. Introduction

Salt stress is becoming a major environmental problem because of the massive crop loss caused by high salinity in soil, especially in arid and semi-arid regions around the world [1]. Salt stress results in ion toxicity, osmotic stress and oxidative stress [2], leading to severe damage to plants. Therefore, it is imperative to improve plant salt tolerances by employing various strategies. Genetic engineering is an effective approach for creating tolerant transgenic plants tolerant to salt stress, and one of the key steps is to identify important salt-responsive genes. To this end, it is pressing and important to identify key genes and unravel their functions in salt tolerance.

Transcription factors are important regulatory proteins that can either activate or inhibit the expression of downstream target genes by binding directly to the promoters [3]. The MYB family transcription factors are widely present in all angiosperms. MYB proteins contain a conserved domain, which usually locates at the N-terminal region [4]. According to the number of MYB domains, MYB transcription factors can be classified into four major subgroups: 1R, R2R3, 3R and 4R-MYB proteins [5]. The R2R3-MYB transcription factors are plant-specific ones with the largest group members, such as 124 members in *Arabidopsis* *thaliana* [6], 192 in *Populus* *trichocarpa* [7], 244 in *Glycine max* [8], and 100 in *Citrus sinensis* [9].

Increasing MYB transcription factors have been identified in the last two decades, and studies showed that MYBs played important roles in multiple plant processes, such as growth and development, phenylpropanoid metabolism, hormone homeostasis, biotic and abiotic stresses responses, and light response [3,4,10,11]. MYB transcription factors are involved in the responses of various abiotic stresses, including drought [12,13], low temperature [14,15,16], high temperature [17] and salt [13,18,19]. For salt stress, MYB transcription factors can play a role in the salt tolerance of plants by directly regulating the expression of downstream target genes. For example, *Arabidopsis* MYB transcription factor AtMyb73 negatively regulates salt tolerance by changing the expression of two salt overly sensitive (SOS) genes, *SOS1* and *SOS3* [20]. AtMYB20 enhances salt tolerance by negatively regulating the gene expression of the type 2C serine/threonine protein phosphatases [21]. The *Arabidopsis* MYB gene, *ATDIV2*, negatively regulates ABA signaling in response to salt stress [22]. Apple (*Malus domestica* Borkh.) MdMYB46 positively regulates salt responsive genes, *RD29A* and *RD22*, to function in the salt tolerance [18]. Some other MYB transcription factors, like TaMYBsdu1, TaMYB3R1 and TaMYB33 from wheat, CmMYB2 from chrysanthemum, MdSIMYB1 from apple, RhMYB96 from rose, have all been proven to function in salt tolerance [19,23,24,25,26,27], suggesting the pivotal role of MYBs from various plants in modulation of salt stress response.

Redox components are involved in all vital activities during plant growth and development [28]. Reactive oxygen species (ROS), such as H_2_O_2_ and O_2_^•−^, act as important signaling molecules in plant cells [29]. However, under abiotic stresses, excessive accumulation of ROS will generate oxidative stress harmful to plants [30]. To cope with this, plants have evolved a sophisticated mechanism to scavenge the harmful ROS by mobilizing a range of antioxidant enzymes, like peroxidase (POD), superoxide dismutase (SOD) and catalase (CAT). In this regard, the regulation of antioxidant enzymes plays a significant role in enhancing plant tolerance to abiotic stresses, as has been revealed in previously studies [31,32,33].

Trifoliate orange (*Poncirus trifoliata* (L.) Raf.) is widely used as rootstock in citrus because of its elite attributes, such as remarkable cold tolerance and desirable disease resistance [34,35]. However, the salt tolerance of trifoliate orange is weak [36], so improving the salt tolerance is a major goal for trifoliate orange, which needs to firstly identify key genes involved in the salt responses. In this study, we identified one R2R3-MYB transcription factor, PtrMYB3, from trifoliate orange, and elucidated its function in salt tolerance. Our findings revealed a negatively regulatory pathway of salt tolerance in trifoliate orange.

## 2. Materials and Methods

### 2.1. Plant Materials and Stress Treatments

To obtain the trifoliate orange (*Poncirus trifoliata* (L.) Raf.) seedlings, seeds collected from trifoliate orange fruits that were harvested in the citrus germplasm repository of Huazhong Agricultural University (Wuhan, China) were sown in commercial soil mixture, and germinated under the natural photoperiod in the greenhouse. Three-month-old seedlings with uniform growth status were used to conduct abiotic stress treatments including salt, dehydration and cold. For salt treatment, seedlings detached from soil were placed in a conical flask with the roots immersed in 250 mM NaCl for 0, 4, 8, 12, 24, 48 and 72 h. For dehydration treatment, seedlings were placed in clean paper under an ambient environment for 0, 1, 3, 6, 12, 24, 48 h, and replaced in water for recovery for 24 h. For cold treatment, seedlings in soil pots were placed in a growth chamber under 4 °C for 0, 6, 24, 72, 120 h, and replaced in 24 °C for 24 h. The leaves were sampled at each designated time point and immediately frozen in liquid nitrogen. All samples were stored in a –80 °C refrigerator for the following analysis.

### 2.2. Quantitative Real-Time Polymerase Chain Reaction (qPCR) Analysis

Total RNA was extracted from the samples stored in −80 °C using a RNAiso Plus kit (TaKaRa, Dalian, China) according to the manufacturer’s instrument. The RNA quality was assessed by gel electrophoresis, and the RNA concentration was measured by a NanoDrop 2000 UV-vis Spectrophotometer (ThermoFisher Scientific, Waltham, MA, USA). The cDNA was synthesized with a RevertAid First Strand cDNA Synthesis Kit (Thermo, Waltham, MA, USA), and cDNA quality was evaluated with the NanoDrop 2000 UV-vis Spectrophotometer. The diluted cDNA was used for qPCR. Primers used for qPCR were designed by the online Primer-blast program in NCBI (https://www.ncbi.nlm.nih.gov/tools/primer-blast/, accessed on 2 June 2020) (Table S1). For qPCR, the 20 μL of reaction mixture consisted of 10 μL of 2 × SYBR Green PCR Master Mix (QIAGEN, Dusseldorf, Germany), 2 μL of ROX dye, 0.25 μM of forward and reverse primers, and 50 ng of cDNA. The qPCR analysis was processed on a QuantStudio 7 Flex (ThermoFisher Scientific, Waltham, MA, USA) system according to the following cycling regimes: 95 °C for 2 min, 95 °C for 10 s (40 cycles), 60 °C for 30 s (40 cycles) and 72 °C for 25 s. The relative gene expression levels were calculated based on the 2^−ΔΔCT^ method [37]. *ACTIN* and *Ubiquitin* were used as internal reference genes for trifoliate orange and tobacco respectively.

### 2.3. Cloning and Sequence Analysis of PtrMYB3

The coding sequence (CDS) of PtrMYB3 (ID: Pt1g018990) from the trifoliate orange genome (http://citrus.hzau.edu.cn/orange/download/index.php, accessed on 7 January 2021) was used to design primers for cloning this gene. Primers were designed with Primer Premier5 software, and primer sequences (PtrMYB3-T) are shown in Table S1. The homologous gene sequences of PtrMYB3 from other plant species were downloaded from NCBI (https://www.ncbi.nlm.nih.gov/, accessed on 16 April 2020). Multiple sequence alignments were performed by ClustalX software based on the protein sequence of PtrMYB3 and the homologous gene sequences of other five species including *Vitis vinifera*, *Oryza sativa*, *Zea mays*, *Brassica rapa*, and *Arabidopsis thaliana*.

### 2.4. Subcellular Localization

For testing the subcellular localization, the CDS of PtrMYB3 without the stop codon was cloned into the *EcorR*I and *BamH*I restriction sites of the 101LYFP vector, which contained a CaMV35S promoter and a yellow fluorescence protein (YFP) reporter gene. The formed construct (PtrMYB3-YFP) and the control vector (YFP without PtrMYB3) were respectively transferred into *Agrobacterium tumefaciens* strain GV3101. Transient expression was performed by co-injecting constructed GV3101 strain and a positive control strain (with a nuclear marker gene *VirD2NLS* fused to mCherry) into the hypodermis of tobacco (*Nicotiana benthamiana*) leaves. The infiltrated tobacco plants were placed in a growth chamber at 25 °C for 3 d under a 16 h light/8 h dark photoperiod. The fluorescence signal was detected and imaged with a laser scanning confocal microscope (Leica TCS-SP8, Frankfurt, Germany).

### 2.5. Transactivation Activity Assay

For analyzing the transactivation activity, the CDS of PtrMYB3 was amplified into the *EcorR*I and *BamH*I restriction sites of the pGBKT7 vector. The fused construct (PtrMYB3-pGBKT7) and the negative control (pGBKT7 without PtrMYB3) were separately transformed into the yeast strain AH109, which harbored three reporter genes including *ADE2*, *HIS3* and *MEL1*. A transactivation assay was performed by incubating the transformed yeast strains on the selective yeast growth mediums, including SD/-Trp, SD/-Trp/-His/-Ade supplemented with 0, 5, 15 mM 3-amino-1,2,4-triazole (3-AT), and SD/-Trp/-His/-Ade supplemented with 20 mM X-α-Gal.

### 2.6. Genetic Transformation of Tobacco

To overexpress PtrMYB3, the full-length CDS was cloned into the *Xba*I and *Sma*I restriction sites of the pBI121 vector under the control of the CaMV35S promoter. The fused vector was transformed into the *A. tumefaciens* GV3101 strain. Genetic transformation of tobacco (*N. tabacum*) was performed using leaf disc as explants according to previous methods [38]. To identify positive transformed seedlings, DNA was firstly extracted from the leaves, and PCR was performed with the extracted DNA and two pairs of specific primers (*NPTⅡ*-F/R and *35S*-F+PtrMYB3-T-R, Table S1). The gene expression levels of PtrMYB3 in the identified transgenic plants were measured by qPCR, according to the methods described above. The transgenic tobacco plants of T0 generation were grown in a growth chamber to harvest seeds and obtain transgenic seedlings of the T1 generation. T1 generation seedlings were conducted positive identification again as described above, and the T2 stable transgenic tobacco plants were used for the subsequent experiments.

### 2.7. Virus Induced Gene Silencing (VIGS)

To obtain PtrMYB3-silenced seedlings, a VIGS system was employed according to the previous study [31]. A 350 bp fragment of PtrMYB3 was inserted into the *BamH*I and *Sma*I restriction sites of the pTRV2 vector. The constructed PtrMYB3-pTRV2, as well as pTRV2 and pTRV1 vectors, was separately transformed into *A. tumefaciens* GV3101 strain. Before agroinfiltration, germinating seeds of trifoliate oranges were prepared in advance, by placing seeds on the soaked gauze for about 10 d. When agroinfiltration was carried out, the bacterial cells were suspended in a 2-(*N*-morpholino) ethanesulfonic acid (MES) buffer to a concentration of OD_600_ = 1, and pTRV1 suspension was mixed with PtrMYB3-pTRV2 or pTRV2 in a ratio of 1:1. The germinated seeds were submerged into the bacterial suspension mixture in vacuum for 30 min. After water cleaning, the seeds were placed in the dark for 3 d. Washed in water for 2–3 times, the seeds were grown in soil for 3–4 weeks. Fully expanded leaves were sampled and DNA was extracted from each plant. Genomic PCR and qPCR were performed to screen positive PtrMYB3-silenced plants.

### 2.8. Salt Tolerance Assay

To investigate the salt tolerance of PtrMYB3 transgenic tobacco plants, the seeds of the T2 generation of two transgenic lines and wild type (WT) were sown in soil pots, with four pots for each line. All the pots were placed in a growth chamber under a 16 h light/8 h dark photoperiod until seeds germinated. The 7-day-old seedlings of every soil pot were sprayed with 20 mL of 200 mM NaCl solution at 3 d intervals two times. The survival rates were calculated after this treatment. In addition, 40-day-old tobacco plants were used to perform salt treatment by spraying and watering with 20 mL of 200 mM NaCl solution at 3 d intervals five times. Three seedlings were planted in one soil pot, with in total eight pots for every line (including the two transgenic lines and one WT line) for salt treatment. Chlorophyll (a, b and total) content of the WT and transgenic tobacco leaves after salt treatment was measured according to the previous method [39].

For the trifoliate oranges of VIGS, one-month-old seedlings were used to perform salt tolerance assays by spraying with 20 mL of 300 mM NaCl solution and watering with 50 mL of 300 mM NaCl solution for every pot at 3 d intervals for 25 d. After treatment, chlorophyll (a, b and total) content of silenced seedlings (PtrMYB3-TRV) and control (WT) were measured as mentioned above. Before and after treatment, the chlorophyll fluorescence was detected with an IMAGING-PAM chlorophyll fluorometer (Walz, Effeltrich, Germany). The maximum quantum efficiency of photosystem II (Fv/Fm) was calculated by an Imaging WinGegE software according to the chlorophyll fluorescence imaging [40].

### 2.9. Dual-Luciferase Reporter Assay

The CDS of PtrMYB3 was amplified and inserted into the *BamH*I and *EcoR*I restriction sites of pGreenII 62-SK vector as effector. The promoter sequence of *PtrPOD* was cloned into the *Hind*III and *BamH*I restriction sites of pGreenII 0800-LUC vector as reporter. The recombinant vectors were transformed into *A. tumefaciens* GV3101 (with a helper plasmid pSoup), and transiently expressed in tobacco (*N. benthamiana*) leaves according to the previous method [31]. A purchased kit of the Dual-Luciferase Reporter Assay System (Promega, Madison, WI, USA) was used to examine the activities of firefly luciferase (LUC) and Renilla luciferase (REN) according to the manufacturer’s instructions with an Infinite 200 Pro microplate reader (Tecan, Switzerland). The LUC/REN ratios were calculated with the control (effector without PtrMYB3) setting as 1. In addition, the transiently expressed tobacco plants were placed in dark for 20 min, and the detached leaves were used to detect and image the luminescence on a NightSHADE LB983 imaging system (Berthold, Stuttgart, Germany).

### 2.10. Hydrogen Peroxide (H_2_O_2_) Measurement

H_2_O_2_ content in tobacco leaves was measured using the purchased Hydrogen Peroxide Assay Kit (Nanjing Jiancheng Bioengineering Institute, Nanjing, China) according to the user manual and the previously described methods [32]. In situ H_2_O_2_ accumulation in tobacco leaves was examined by histochemical staining with 3,3′-diaminobenzidine (DAB). Leaves were immersed in 1 mg/mL DAB solution (prepared with pH 7.8 phosphatic buffer solution, adding 30% H_2_O_2_ before staining), and put in room temperature for staining for 1–2 d. The stained leaves were discolored with 80% ethanol under 60 °C, and photographed after drying.

### 2.11. Statistical Analysis

All the data, shown as means ± SE, were analyzed by the SAS software (SAS Institute, Cary, NC, USA) and Microsoft Office Excel. The significance of statistical difference was analyzed by SPSS software (IBM, Chicago, IL, USA) based on the Fisher’s LSD test in the one-way analysis of variance (ANOVA) program, taking *p* < 0.05 (*), *p* < 0.01 (**) and *p* < 0.001 (***) as significance levels.

## 3. Results

### 3.1. Cloning and Sequence Analysis of PtrMYB3

**Figure 1:**
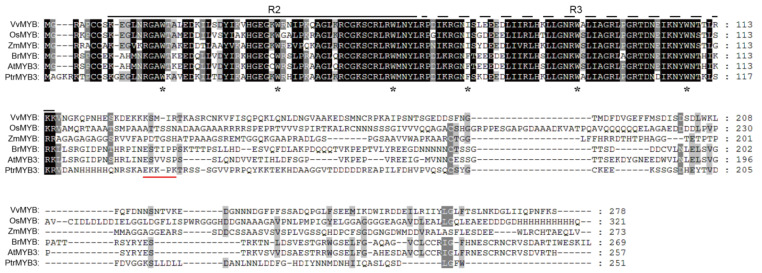
Multiple sequence alignment of PtrMYB3 and another five *R2R3-MYB* genes from five species, including *Vitis vinifera* (VvMYB, CBI20715.3), *Oryza sativa* (OsMYB, XP_015629423.1), *Zea mays* (ZmMYB, NP_001358856.1), *Brassica rapa* (BrMYB, XP_009115618.1), *Arabidopsis thaliana* (AtMYB, NP_564176.2). The R2 and R3 MYB domains are indicated with a black solid line and a dotted line, respectively. The conserved tryptophan (W) residues are indicated with asterisks (*). A nuclear localization signal (NLS) is indicated with a red solid line.

From our previous RNA-sequencing data [36], a gene (gene ID: Pt1g018990) was found significantly upregulated after salt stress treatment in trifoliate orange. To study this gene, we cloned its ORF (open reading frame) sequence, which was 756 bp encoding a putative protein of 251 amino acids (aa). By BLAST analysis in the *Arabidopsis* genome database, the protein sequence of this gene showed the highest identity with AtMYB3 (AT1G22640), and one R2R3-MYB transcription factor. The gene was designated as PtrMYB3 (*Poncirus trifoliata MYB3*). By sequence alignment with the homologous genes from other five plant species (*Vitis vinifera*, *Oryza sativa*, *Zea mays*, *Brassica rapa*, and *Arabidopsis thaliana*), PtrMYB3 was found possessing a conserved R2R3-MYB domain in the N-terminal region and a quite non-conservative C-terminal region (Figure 1). In addition, the N-terminal region of PtrMYB3 contained a short sequence of nuclear localization signal (NLS). Cloning and sequence analysis showed that PtrMYB3 is a typical R2R3-MYB transcription factor.

### 3.2. PtrMYB3 Is Induced by Multiple Abiotic Stresses

**Figure 2:**
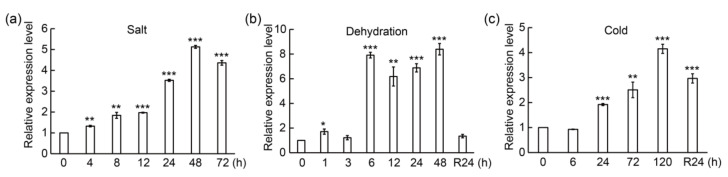
Relative expression levels of PtrMYB3 under salt (**a**), dehydration (**b**) and cold (**c**) stresses at each designated time point. Error bars indicate ± SE (standard error). R: recovery. Asterisks indicate significant differences compared to the value at 0 h: *p* < 0.05 (*), *p* < 0.01 (**) and *p* < 0.001 (***).

To ascertain the function of PtrMYB3, real-time quantitative PCR (qPCR) was used to measure the expression levels under abiotic stresses including salt, dehydration and cold. The results showed that the relative expression level of PtrMYB3 was continuously increased within 48 h of salt treatment (Figure 2a). Under dehydration treatment, the expression of PtrMYB3 was maintained stable during the first 3 h treatment, sharply reached the top at 6 h treatment, and reverted to the basal level after recovery treatment for 24 h (Figure 2b). Under cold stress, PtrMYB3 was gradually induced within the 120 h treatment, and then slightly decreased after recovery for 24 h (Figure 2c). These results reveal that PtrMYB3 can be induced by various abiotic stresses.

### 3.3. PtrMYB3 Is Localized to Nucleus

**Figure 3:**
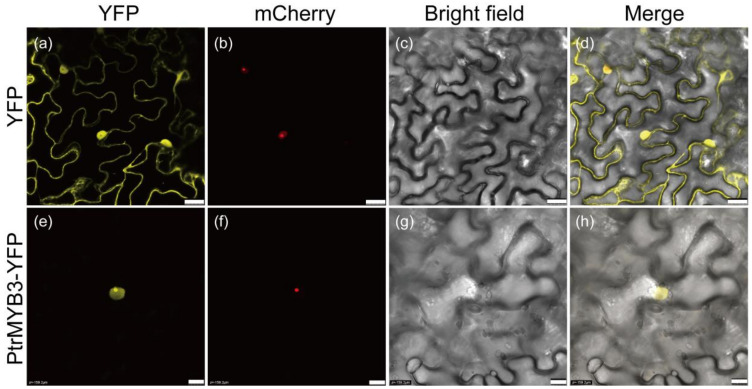
Subcellular localization of PtrMYB3. The representative images indicate cells expressing YFP (**a**–**d**) or PtrMYB3-YFP fusion protein (**e**–**h**) under different fields, including yellow fluorescence (YFP, (**a**,**e**)), red fluorescence (mCherry, (**b**,**f**)), bright field (**c**,**g**) and merged field (**d**,**h**). The red fluorescence images (**b**,**f**) show the signal of a nuclear marker gene *VirD2NLS* fused to mCherry. Bar, shown at the right bottom, =25 μm (upper panels) and 10 μm (bottom panels).

A nuclear localization signal was found in the N-terminal region of PtrMYB3 protein (Figure 1), suggesting that this protein might be localized to the nucleus. To test its subcellular localization, the full-length coding sequence of PtrMYB3 was fused to a yellow fluorescence protein (YFP) driven by the cauliflower mosaic virus 35S (CaMV35S) promoter. The fused protein (PtrMYB3-YFP), as well as the control unfused protein (YFP), was transiently expressed in the epidermal cells of tobacco leaves. One nuclear-localized marker gene fused to mCherry (with red fluorescence) was co-transformed. Observed in the imaging of the confocal microscopy, the yellow fluorescence signal of the fused protein (PtrMYB3-YFP) was exclusively detected in the nuclear, the same as the mCherry signal of the nuclear-localized marker gene, while the YFP signal of the unfused protein was found throughout the whole cell including the nucleus and cytoplasm (Figure 3), indicating that PtrMYB3 is a nuclear-localized protein.

### 3.4. PtrMYB3 Has Transactivation Activity

**Figure 4:**
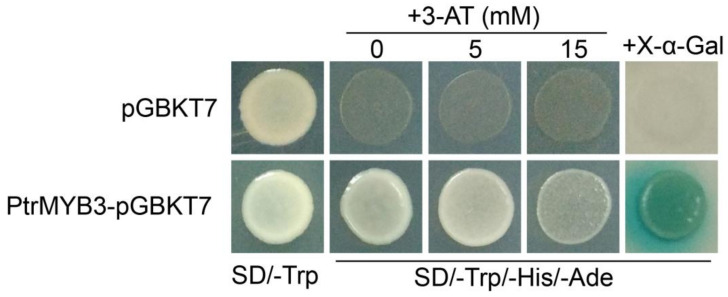
Transactivation assay of PtrMYB3. The representative images indicate yeast cells expressing pGBKT7 control plasmid (upper panels) or PtrMYB3-pGBKT7 fusion plasmid (bottom panels) under different selective growth mediums, including SD/-Trp or SD/-Trp/-His/-Ade with or without 3-AT and X-α-Gal.

As a transcription factor, possessing transactivation activity is a defining feature for PtrMYB3. To verify this, the full-length coding sequence of PtrMYB3 was fused to a GAL4 DNA-binding domain in the pGBKT7 vector, and the fused construct (PtrMYB3-pGBKT7), as well as the control empty vector (pGBKT7), was transformed separately into the yeast strain AH109, which carried reporter genes including *ADE2*, *HIS3* and *MEL1*. The transactivation activity was tested by the observation of the yeast growth under selective growth mediums. The results showed that the yeast cells transformed either control vector (pGBKT7) or fused constructs (PtrMYB3-pGBKT7) could grow well on the SD/-Trp medium (Figure 4), indicating that the transformation system was reliable. On the selective SD/-Trp/-His/-Ade medium, only the yeast cells expressing PtrMYB3-pGBKT7 could survive, and the survived yeast colony turned blue when supplemented with X-α-Gal (Figure 4), indicating that PtrMYB3 transcription factor has transactivation activity.

### 3.5. Overexpression of PtrMYB3 Impairs the Salt Tolerance of Tobacco

**Figure 5:**
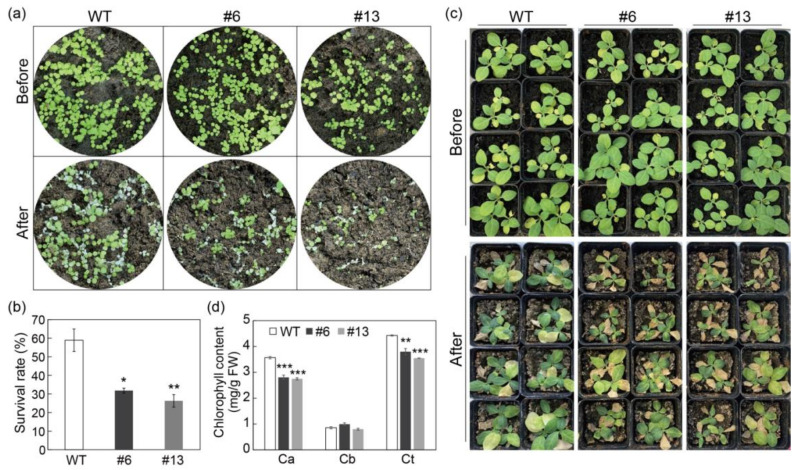
Salt tolerance assays of transgenic tobacco plants overexpressing PtrMYB3. (**a**) Representative images of 7-day-old tobacco plants of the two transgenic lines (#6 and #13) and wild-type (WT) before and after salt treatment. (**b**) The survival rate of the two transgenic lines and WT after salt treatment. (**c**) Representative images of 40-day-old tobacco plants of the two transgenic lines and WT before and after salt treatment. (**d**) The chlorophyll content (a, b and total) of the two transgenic lines and WT after salt treatment. Ca: chlorophyll a; Cb: chlorophyll b; Ct: total chlorophyll including a and b. Error bars indicate ± SE. Asterisks indicate significant differences between the transgenic line and WT: *p* < 0.05 (*), *p* < 0.01 (**) and *p* < 0.001 (***).

As PtrMYB3 was strongly induced by salt stress (Figure 2a), it was speculated to play a role in salt stress tolerance. To investigate its putative function, transgenic tobacco overexpressing PtrMYB3, under the control of CaMV35S promoter, was generated by *Agrobacterium*-mediated transformation (Figure S1a). Positive identification was conducted with two pairs of primers, and 14 transgenic tobacco lines were finally screened (Figure S1b), among which two lines (#6 and #13) showed significantly elevated expression of PtrMYB3 (Figure S1c). With the T2 generation of these two lines and one wild-type (WT) line, 7-day-old potted seedlings were conducted salt treatment by spraying 200 mM NaCl solution. Before treatment, there was no morphological difference between the transgenic lines and WT. After treatment, all lines had some dead plants with chlorosis phenotype, while this damage was visibly more severe in the transgenic plants in comparison with the WT plants (Figure 5a), with significantly lower survival rate in two transgenic lines than in the WT (Figure 5b). In addition, to further confirm the salt tolerance of adult plants, 40-day-old seedlings of transgenic lines and WT were used to perform salt treatment with 200 mM NaCl solution. Before stress treatment, no conspicuous difference was detected between the transgenic lines and WT. After stress, the two transgenic lines exhibited more serious leaf damage in comparison with the WT plants (Figure 5c), concomitant with significantly lower chlorophyll content (chlorophyll a and total) in two transgenic lines than in the WT (Figure 5d). These results indicate that overexpression of PtrMYB3 in tobacco impairs its salt tolerance.

### 3.6. Silencing of PtrMYB3 in Trifoliate Orange Confers Enhanced Salt Tolerance

**Figure 6:**
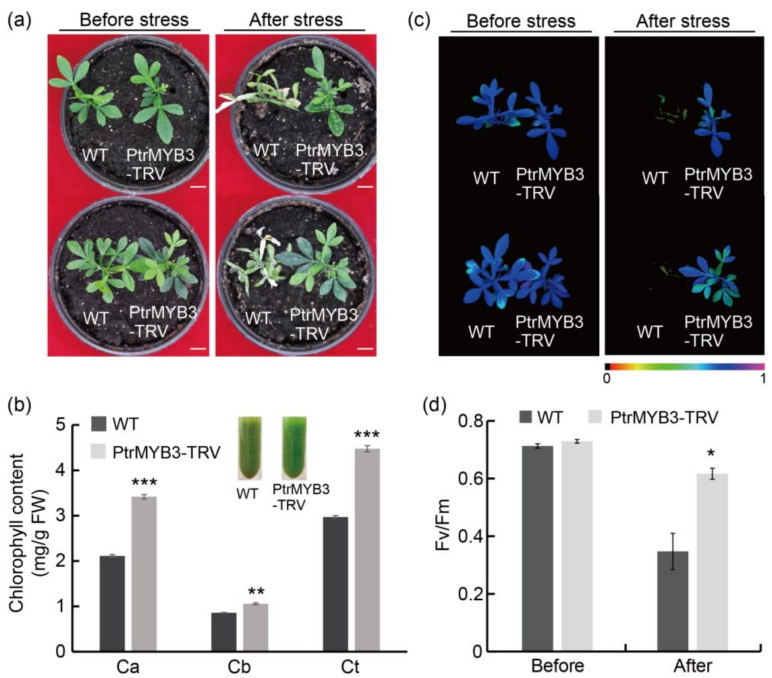
Silencing of PtrMYB3 in *Poncirus trifoliata* confers enhanced salt tolerance. (**a**) Phenotypes of WT and PtrMYB3-silenced plants (PtrMYB3-TRV) before and after salt stress. Bar = 1 cm. (**b**) Chlorophyll content (a, b and total) of WT and *PtrMYB3-*silenced plants after salt stress. The images above show the crude extract in WT and PtrMYB3-silenced plants. (**c**,**d**) The chlorophyll fluorescence imaging (**c**) and the Fv/Fm values (**d**) of WT and PtrMYB3-silenced plants before and after salt stress. Error bars indicate ± SE. Asterisks indicate significant differences between WT and PtrMYB3-silenced plants: *p* < 0.05 (*), *p* < 0.01 (**) and *p* < 0.001 (***).

Overexpression of PtrMYB3 in tobacco confirmed the negatively regulated role of this gene in salt tolerance. To further verify this function, a virus-induced gene silencing (VIGS) system was employed to obtain PtrMYB3-silenced trifoliate orange seedlings (Figure S2a). Transcript abundance of PtrMYB3 in the silenced seedlings was repressed, to varying degrees, in comparison with the control seedlings (WT) (Figure S2b). With the obtained silenced seedlings (PtrMYB3-TRV) and WT, a salt stress assay was conducted by watering and spraying 300 mM NaCl solution. Before stress, the silenced seedlings and WT showed no morphological difference. However, after stress, WT leaves exhibited obviously wilting and yellowing while the silenced plants still grew well with totally green leaves (Figure 6a). Consistent with the morphological phenotype, chlorophyll (a, b and total) content in the leaves of silenced plants was significantly higher than that in the WT leaves (Figure 6b). In addition, chlorophyll fluorescence imaging, which could reflect the growth status by monitoring the photosynthesis, showed no difference between WT and the silenced lines before salt stress treatment, whereas after salt stress, WT plants exhibited more serious photosynthetic damage compared with the silenced plants (Figure 6c). Meanwhile, coupled with the chlorophyll fluorescence imaging, the maximum quantum efficiency of photosystem II (Fv/Fm) in silenced plants was significantly higher than that in WT plants after stress (Figure 6d). Taken together, these data demonstrate that silencing of PtrMYB3 in trifoliate orange confers enhanced salt tolerance, and PtrMYB3 is a negative regulator of salt tolerance.

### 3.7. PtrMYB3 Negatively Regulates the Expression of a Peroxidase Gene, PtrPOD

**Figure 7:**
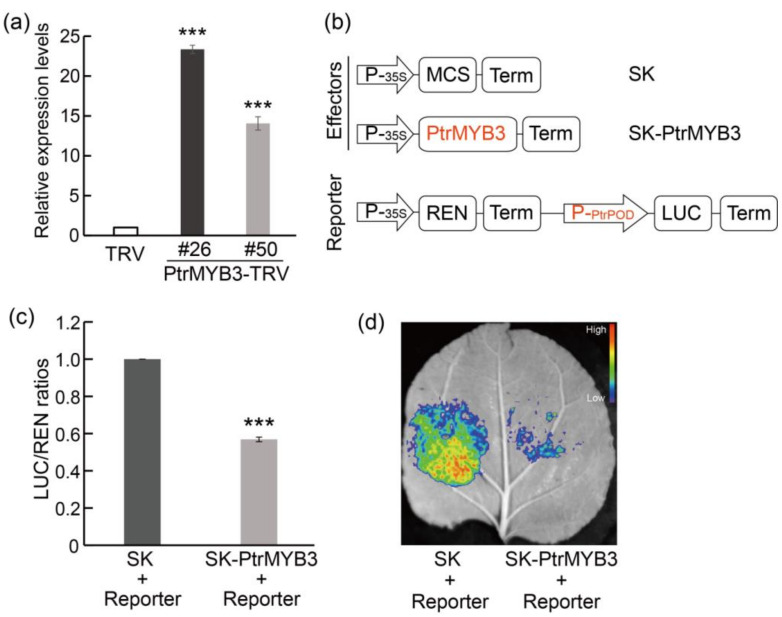
PtrMYB3 negatively regulates the expression of *PtrPOD*. (**a**) The relative expression levels of *PtrPOD* in WT and two PtrMYB3-silenced lines (#26 and #50). (**b**) Schematic diagrams of the reporter and the effectors used for dual-luciferase activity assay. P-_35S_: CaMV35S promoter; Term: terminator; MCS: multiple cloning sites; REN: renilla luciferase; LUC: firefly luciferase; P-_PtrPOD_: the promoter of *PtrPOD*; SK: pGreenII 62-SK vector. (**c**) The relative LUC/REN ratios in tobacco leaves transiently co-expressing the reporter and the effector. The LUC/REN ratio of empty vector (SK) without PtrMYB3 is set to 1. (**d**) The representative image of one tobacco leaf transiently co-expressing the reporter and the effector. The fluorescence intensity is indicated with different colors from low to high as shown in the color scale bar. Error bars indicate ± SE. Asterisks indicate significant differences: *p* < 0.001 (***).

To study the underlying mechanism of PtrMYB3-regulated salt sensitivity, we examined the expression of the salt stress-related genes in PtrMYB3-silenced trifoliate orange, and a peroxidase gene (named as *PtrPOD*, ID: Pt6g017020) was finally found to be remarkably up-regulated in two PtrMYB3-silenced lines in comparison with the WT (Figure 7a). Peroxidase, which functions in eliminating excessive reactive oxygen species (ROS), plays a vital role in the stress tolerance of plants [41]. Therefore, it was speculated that PtrMYB3 might regulate salt sensitivity by negatively regulating the gene expression of *PtrPOD*. To verify this assumption, a dual-luciferase reporter system was employed by constructing “Effector” (SK-PtrMYB3 or SK) and “Reporter” (inserting *PtrPOD* promoter upstream the LUC reporter gene) (Figure 7b). Both the effector and the reporter were co-expressed transiently in tobacco leaves, and the promoter activity of *PtrPOD* with or without PtrMYB3 was tested by the measurement of a relative LUC/REN ratio. The results showed that the LUC/REN ratio was significantly lower in the presence of PtrMYB3 than the control without PtrMYB3 (Figure 7c), indicating that the presence of PtrMYB3 can suppress the promoter activity of *PtrPOD*. Consistent with the LUC/REN ratio, in vivo fluorescence imaging showed an obviously weaker fluorescence when PtrMYB3 was expressed than when PtrMYB3 was absent (Figure 7d). These data reveal that PtrMYB3 serves as a negative regulator and can suppress the expression of *PtrPOD*.

### 3.8. PtrMYB3 Transgenic Tobacco Plants Accumulated Excess H_2_O_2_ after Salt Stress

**Figure 8:**
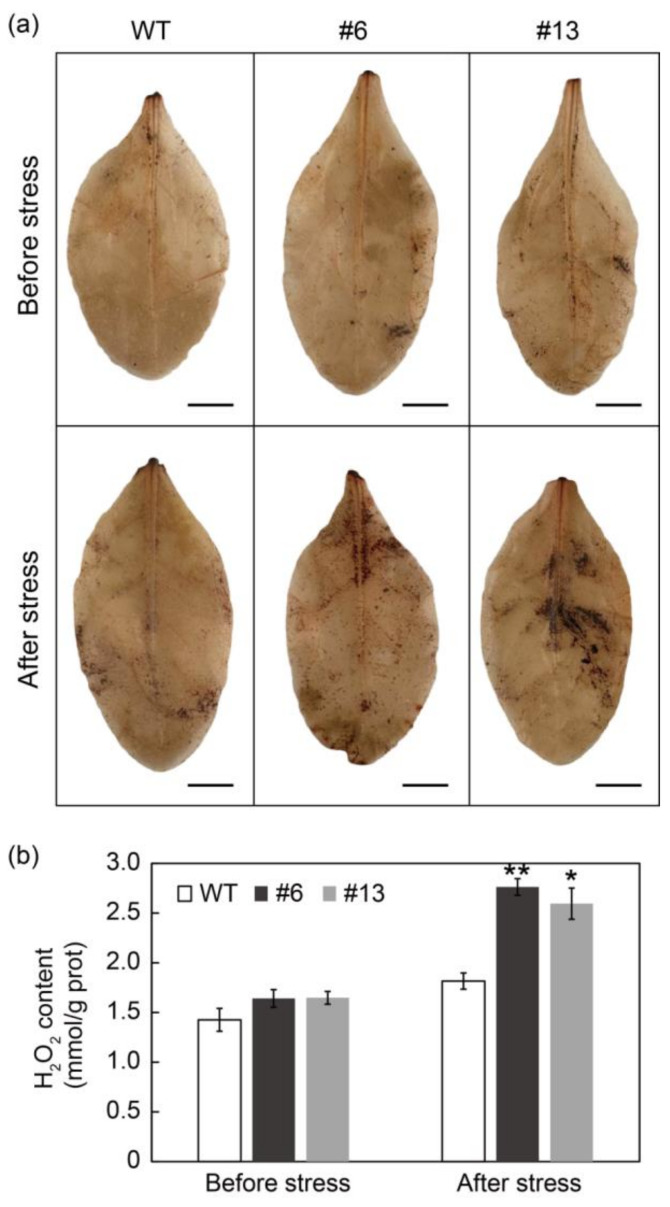
PtrMYB3 transgenic tobacco plants accumulate excess H_2_O_2_ after salt stress. (**a**) In situ H_2_O_2_ staining with 3,3′-diaminobenzidine (DAB) in the leaves of two transgenic lines (#6 and #13) and the WT before and after salt stress. Bar = 1 cm. (**b**) H_2_O_2_ content measurement in the leaves of two transgenic lines (#6 and #13) and the WT before and after salt stress. Error bars indicate ± SE. Asterisks indicate significant differences: *p* < 0.05 (*), *p* < 0.01 (**).

As *PtrPOD*-encoded peroxidase functions in the scavenging of H_2_O_2_ under abiotic stresses, the H_2_O_2_ accumulation in transgenic tobacco plants overexpressing PtrMYB3 was examined before and after salt stress (Figure 8). In situ H_2_O_2_ staining with 3,3′-diaminobenzidine (DAB) showed that the leaves of transgenic lines (#6 and #13) and WT both had no staining before salt stress, while transgenic lines had obviously darker and more extensive staining than the WT after salt stress (Figure 8a), indicating that PtrMYB3 transgenic plants accumulated more H_2_O_2_ than the WT after salt stress. Further quantitative measurement of H_2_O_2_ in the leaves of transgenic tobacco plants and WT presented the same results, which showed that the H_2_O_2_ content in the transgenic lines and WT had no significant differences before salt stress, while after stress, H_2_O_2_ content was increased in all tobacco plants, and the two transgenic lines had significantly higher content than the WT (Figure 8b). These results demonstrate that transgenic tobacco plants overexpressing PtrMYB3 accumulate excess H_2_O_2_ after salt stress.

## 4. Discussion

Salt stress can generate osmotic stress, ion toxicity and oxidative stress, resulting in severe damage to plants [2]. Trifoliate orange is one of the most commonly used rootstocks in citrus, but its extensive application is limited by the salt sensitivity [34,35]. It is of great significance to study the mechanisms underlying the responses of trifoliate orange to salt stress, mainly by discovering important responsive genes and revealing their functions in salt tolerance. The MYB transcription factors, which are widely present in plants, participate in a variety of physiological processes in plant life [10]. Increasing studies have shown that MYB transcription factors are also involved in the response to abiotic stresses [4,10,42]. In this study, a gene encoding R2R3-MYB transcription factor, designated as PtrMYB3, was cloned from trifoliate orange, and functional analyzing indicated that this gene negatively regulated salt tolerance. This study can help us better understand the response mechanisms of trifoliate orange to salt stress.

### 4.1. PtrMYB3 Is Induced by Various Stresses

Through the expression profiling under different abiotic stresses, PtrMYB3 was found to be able to be induced by salt, dehydration, and cold stresses, indicating that this gene may be involved in a variety of stress responses. In this study, we have shown that this gene is involved in salt stress, so the next step is to investigate whether it is also involved in dehydration and cold stresses. Previous studies also indicated that one same MYB transcription factor could be involved in multiple abiotic stresses simultaneously [4,26]. For example, ZmMYB3R was found to be involved in both drought and salt stresses in maize (*Zea mays* L.) [13]. MYB3R from wheat (*Triticum aestivum* L.) functioned in drought, salt and cold stresses [26]. Two MYB transcription factors from rose (*Rosa rugosa*) were identified involved in both mechanical injury and oxidative stress [43]. This may be explained by the interconnection between different stresses. When a MYB transcription factor simultaneously regulates multiple stress responsive genes, or regulates a gene functioning in multiple stresses, this MYB transcription factor will be able to participate in the responses of multiple stresses. In this study, we found that PtrMYB3 could regulate *PtrPOD*, a gene encoding an antioxidant enzyme, which plays a role in a variety of abiotic stresses [44]. Therefore, it is reasonable that PtrMYB3 may function in a variety of abiotic stresses, but more experiments need to be carried out to verify this hypothesis.

### 4.2. PtrMYB3 Negatively Regulates Salt Tolerance

The involvement of MYB transcription factors in response to abiotic stresses has been extensively reported [4], especially in the past decade, with the completion of genome sequencing of many plant species. A large number of MYB transcription factors have been discovered, and their roles in abiotic stresses have been investigated well. In these studies, most MYB transcription factors are involved in stress responses as positive regulators, which play a positive role in salt tolerance [14,18,45], while few negative regulators have been discovered. In previous reports, some MYB transcription factors were found negatively regulating fruit color [46] and fruit ripening [47,48], but few were found in abiotic stresses, except for one MYB transcription factor identified from rice which negatively regulated cold tolerance [49]. In this study, one R2R3-MYB transcription factor (PtrMYB3) was identified from trifoliate orange as a negative regulator for salt tolerance, which is meaningful for the studies on transcriptional regulation of abiotic stresses.

Transcription factors can be classified into two types: activator and repressor, which play positive and negative regulatory roles on downstream genes respectively [3]. The transcription activator is positively correlated with the expression of downstream genes, and plays the same functions as downstream genes. In contrast, the transcription repressor is negatively correlated with downstream gene expression and plays a possible role in reducing gene expression in order to avoid the possible adverse effect of overexpression. In this study, we found that PtrMYB3 negatively regulates the salt tolerance of trifoliate orange, suggesting that PtrMYB3 might play a role in balancing gene expression and salt tolerance. To be specific, at the early stage of salt stress, a series of responsive genes are induced which might generate the opposite effects on plants. In this situation, transcription repressors can play a crucial role by decreasing the expression of some responsive genes. After stress for a long time, the salt responsive genes are still maintained in a state of high expression that will inevitably cause adverse effects, so transcription repressors are required to orchestrate a dynamic expression for most genes. In this study, PtrMYB3 was found to be up-regulated at the early stage of salt stress, indicating that PtrMYB3 works as a repressor by suppressing early salt-responsive genes, including *PtrPOD*, causing salt sensitivity of trifoliate orange, which is generally considered as a salt-sensitive species in citrus. In a word, transcription repressors are also very significant players for governing plant responses to the abiotic stresses.

### 4.3. PtrMYB3 Regulates *PtrPOD*-Mediated H_2_O_2_ Accumulation

In this study, *PtrPOD* expression was found to be significantly increased when PtrMYB3 was silenced in trifoliate orange, and the dual-luciferase (LUC) assay further confirmed that PtrMYB3 could suppress the expression of *PtrPOD* promoter (Figure 7), suggesting that PtrMYB3 can regulate *PtrPOD* expression. *PtrPOD* encodes peroxidase (POD), which is an important antioxidant enzyme functioning in scavenging excessive reactive oxygen species (ROS) accumulated in abiotic stresses [44]. ROS, such as H_2_O_2_, O_2_^•−^, NO, work as intracellular signaling molecules in an appropriate concentration range. However, under abiotic stresses, ROS will accumulate sharply, resulting in oxidative stress, which will finally have harmful effects on plant growth [41]. The function of POD in moderating excessive ROS accumulation has been well studied, but the transcriptional regulatory mechanism has not been thoroughly studied. In recent years, some transcription factors have been proved to be able to directly bind to the *POD* promoter to positively regulate its expression under abiotic stresses, such as bHLH18 [32], ERF109 [31], ABF [33], and PtrbHLH [50]. These findings revealed the function of the “transcription factors-*POD*” regulatory module in abiotic stresses. However, the negative regulators of *POD* have not been reported yet. The PtrMYB3, found in this study as a rare transcriptional repressor regulating *POD*, will greatly enrich the transcriptional regulatory network of *POD*. As the H_2_O_2_ accumulation in transgenic tobacco plants overexpressing PtrMYB3 was found significantly increased after salt stress in comparison with the WT (Figure 8), we conclude that PtrMYB3 negatively regulates the expression of the *POD* gene, inhibits peroxidase-mediated H_2_O_2_ scavenging, leads to excess accumulation of H_2_O_2_, and finally impairs the salt tolerance of plants.

## 5. Conclusions

In this study, a MYB transcription factor, PtrMYB3, was identified from trifoliate orange, and its function in salt tolerance was investigated. Studies showed that PtrMYB3 was a typical R2R3-MYB transcription factor, and could be induced by salt, dehydration and cold stresses. Overexpression of PtrMYB3 in tobacco conferred impaired salt tolerance and, by contrast, silencing of this gene by VIGS in trifoliate orange conferred enhanced salt tolerance, indicating that PtrMYB3 negatively regulated salt tolerance. Additionally, a peroxidase gene (*PtrPOD*) was identified as the potential target gene of PtrMYB3, and PtrMYB3 could negatively regulate salt tolerance, at least in part, through the regulation of *PtrPOD*-mediated ROS scavenging.

## Data Availability

Data are contained within the article and supplementary material.

## Supplementary Materials

The following documents are available online at https://www.mdpi.com/article/10.3390/antiox10091388/s1. Figure S1: Genetic transformation and molecular identification of PtrMYB3 overexpressed tobacco plants. Figure S2: Molecular identification of PtrMYB3-silenced *Poncirus trifoliata* plants by genomic PCR and qPCR. Table S1: Primer sequences used in this study.

## References

[1] van Zelm E., Zhang Y., Testerink C. (2020). Salt tolerance mechanisms of plants. Annu. Rev. Plant Biol..

[2] Zhu J.-K. (2016). Abiotic stress signaling and responses in plants. Cell.

[3] Liu J.-H., Peng T., Dai W. (2014). Critical cis-acting elements and interacting transcription factors: Key players associated with abiotic stress responses in plants. Plant Mol. Biol. Rep..

[4] Li C., Ng C.K.Y., Fan L.-M. (2015). MYB transcription factors, active players in abiotic stress signaling. Environ. Exp. Bot..

[5] Prouse M.B., Campbell M.M. (2012). The interaction between MYB proteins and their target DNA binding sites. Biochim. Biophys. Acta.

[6] Chen Y., Yang X., He K., Liu M., Li J., Gao Z., Lin Z., Zhang Y., Wang X., Qiu X. (2006). The MYB transcription factor superfamily of *Arabidopsis*: Expression analysis and phylogenetic comparison with the rice MYB family. Plant Mol. Biol..

[7] Wilkins O., Nahal H., Foong J., Provart N.J., Campbell M.M. (2009). Expansion and diversification of the *Populus* R2R3-MYB family of transcription factors. Plant Physiol..

[8] Du H., Yang S.-S., Liang Z., Feng B.-R., Liu L., Huang Y.-B., Tang Y.-X. (2012). Genome-wide analysis of the MYB transcription factor superfamily in soybean. BMC Plant Biol..

[9] Liu C., Wang X., Xu Y., Deng X., Xu Q. (2014). Genome-wide analysis of the R2R3-MYB transcription factor gene family in sweet orange (*Citrus sinensis*). Mol. Biol. Rep..

[10] Ambawat S., Sharma P., Yadav N.R., Yadav R.C. (2013). MYB transcription factor genes as regulators for plant responses: An overview. Physiol. Mol. Biol. Plants.

[11] Dubos C., Stracke R., Grotewold E., Weisshaar B., Martin C., Lepiniec L. (2010). MYB transcription factors in *Arabidopsis*. Trends Plant Sci..

[12] Zhang S., Feng M., Chen W., Zhou X., Lu J., Wang Y., Li Y., Jiang C.-Z., Gan S.-S., Ma N. (2019). In rose, transcription factor PTM balances growth and drought survival via PIP2;1 aquaporin. Nat. Plants.

[13] Wu J., Jiang Y., Liang Y., Chen L., Chen W., Cheng B. (2019). Expression of the maize MYB transcription factor ZmMYB3R enhances drought and salt stress tolerance in transgenic plants. Plant Physiol. Biochem..

[14] An J.-P., Wang X.-F., Zhang X.-W., Xu H.-F., Bi S.-Q., You C.-X., Hao Y.-J. (2020). An apple MYB transcription factor regulates cold tolerance and anthocyanin accumulation and undergoes MIEL1-mediated degradation. Plant Biotechnol. J..

[15] Xing C., Liu Y., Zhao L., Zhang S., Huang X. (2019). A novel MYB transcription factor regulates ascorbic acid synthesis and affects cold tolerance. Plant Cell Environ..

[16] An J.-P., Li R., Qu F.-J., You C.-X., Wang X.-F., Hao Y.-J. (2018). R2R3-MYB transcription factor MdMYB23 is involved in the cold tolerance and proanthocyanidin accumulation in apple. Plant J..

[17] Liao C., Zheng Y., Guo Y. (2017). MYB30 transcription factor regulates oxidative and heat stress responses through ANNEXIN-mediated cytosolic calcium signaling in *Arabidopsis*. New Phytol..

[18] Chen K., Song M., Guo Y., Liu L., Xue H., Dai H., Zhang Z. (2019). MdMYB46 could enhance salt and osmotic stress tolerance in apple by directly activating stress-responsive signals. Plant Biotechnol. J..

[19] Jiang X., Li S., Ding A., Zhang Z., Hao Q., Wang K., Liu Q., Liu Q. (2018). The novel rose MYB transcription factor RhMYB96 enhances salt tolerance in transgenic *Arabidopsis*. Plant Mol. Biol. Rep..

[20] Kim J.H., Nguyen N.H., Jeong C.Y., Nguyen N.T., Hong S.-W., Lee H. (2013). Loss of the R2R3 MYB, AtMyb73, causes hyper-induction of the *SOS1* and *SOS3* genes in response to high salinity in *Arabidopsis*. J. Plant Physiol..

[21] Cui M.H., Yoo K.S., Hyoung S., Nguyen H.T.K., Kim Y.Y., Kim H.J., Ok S.H., Yoo S.D., Shin J.S. (2013). An *Arabidopsis* R2R3-MYB transcription factor, AtMYB20, negatively regulates type 2C serine/threonine protein phosphatases to enhance salt tolerance. FEBS Lett..

[22] Fang Q., Wang Q., Mao H., Xu J., Wang Y., Hu H., He S., Tu J., Cheng C., Tian G. (2018). AtDIV2, an R-R-type MYB transcription factor of *Arabidopsis*, negatively regulates salt stress by modulating ABA signaling. Plant Cell Rep..

[23] Wang R.-K., Cao Z.-H., Hao Y.-J. (2014). Overexpression of a R2R3 MYB gene *MdSIMYB1* increases tolerance to multiple stresses in transgenic tobacco and apples. Physiol. Plant..

[24] Shan H., Chen S., Jiang J., Chen F., Chen Y., Gu C., Li P., Song A., Zhu X., Gao H. (2012). Heterologous expression of the chrysanthemum R2R3-MYB transcription factor CmMYB2 enhances drought and salinity tolerance, increases hypersensitivity to ABA and delays flowering in *Arabidopsis thaliana*. Mol. Biotechnol..

[25] Qin Y., Wang M., Tian Y., He W., Han L., Xia G. (2012). Over-expression of *TaMYB33* encoding a novel wheat MYB transcription factor increases salt and drought tolerance in *Arabidopsis*. Mol. Biol. Rep..

[26] Cai H., Tian S., Liu C., Dong H. (2011). Identification of a *MYB3R* gene involved in drought, salt and cold stress in wheat (*Triticum aestivum* L.). Gene.

[27] Rahaie M., Xue G.-P., Naghavi M.R., Alizadeh H., Schenk P.M. (2010). A MYB gene from wheat (*Triticum aestivum* L.) is up-regulated during salt and drought stresses and differentially regulated between salt-tolerant and sensitive genotypes. Plant Cell Rep..

[28] Ahmad P. (2016). Water Stress and Crop Plants: A Sustainable Approach.

[29] De Rossi S., Di Marco G., Bruno L., Gismondi A., Canini A. (2021). Investigating the drought and salinity effect on the redox components of *Sulla Coronaria* (L.) Medik. Antioxidants.

[30] Hossain M.S., ElSayed A.I., Moore M., Dietz K.J. (2017). Redox and reactive oxygen species network in acclimation for salinity tolerance in sugar beet. J. Exp. Bot..

[31] Wang M., Dai W., Du J., Ming R., Dahro B., Liu J.-H. (2019). ERF109 of trifoliate orange (*Poncirus trifoliata* (L.) Raf.) contributes to cold tolerance by directly regulating expression of *Prx1* involved in antioxidative process. Plant Biotechnol. J..

[32] Geng J., Liu J.-H. (2018). The transcription factor CsbHLH18 of sweet orange functions in modulation of cold tolerance and homeostasis of reactive oxygen species by regulating the antioxidant gene. J. Exp. Bot..

[33] Zhang Q., Wang M., Hu J., Wang W., Fu X., Liu J.-H. (2015). PtrABF of *Poncirus trifoliata* functions in dehydration tolerance by reducing stomatal density and maintaining reactive oxygen species homeostasis. J. Exp. Bot..

[34] Huang Y., Xu Y., Jiang X., Yu H., Jia H., Tan C., Hu G., Hu Y., Rao M.J., Deng X. (2021). Genome of a citrus rootstock and global DNA demethylation caused by heterografting. Hortic. Res..

[35] Peng Z., Bredeson J.V., Wu G.A., Shu S., Rawat N., Du D., Parajuli S., Yu Q., You Q., Rokhsar D.S. (2020). A chromosome-scale reference genome of trifoliate orange (*Poncirus trifoliata*) provides insights into disease resistance, cold tolerance and genome evolution in *Citrus*. Plant J..

[36] Wei T., Wang Y., Liu J.-H. (2020). Comparative transcriptome analysis reveals synergistic and disparate defense pathways in the leaves and roots of trifoliate orange (*Poncirus trifoliata*) autotetraploids with enhanced salt tolerance. Hortic. Res..

[37] Livak K.J., Schmittgen T.D. (2001). Analysis of relative gene expression data using real-time quantitative PCR and the 2^−ΔΔCT^ method. Methods.

[38] Horsch R.B., Fry J.E., Hoffmann N.L., Eichholtz D., Rogers S.G., Fraley R.T. (1985). A simple and general method for transferring genes into plants. Science.

[39] Dai W., Wang M., Gong X., Liu J.-H. (2018). The transcription factor FcWRKY40 of *Fortunella crassifolia* functions positively in salt tolerance through modulation of ion homeostasis and proline biosynthesis by directly regulating *SOS2* and *P5CS1* homologs. New Phytol..

[40] Wei T., Wang Y., Xie Z., Guo D., Chen C., Fan Q., Deng X., Liu J.-H. (2019). Enhanced ROS scavenging and sugar accumulation contribute to drought tolerance of naturally occurring autotetraploids in *Poncirus trifoliata*. Plant Biotechnol. J..

[41] Choudhury F.K., Rivero R.M., Blumwald E., Mittler R. (2017). Reactive oxygen species, abiotic stress and stress combination. Plant J..

[42] Albert N.W., Thrimawithana A.H., McGhie T.K., Clayton W.A., Deroles S.C., Schwinn K.E., Bowman J.L., Jordan B.R., Davies K.M. (2018). Genetic analysis of the liverwort *Marchantia polymorpha* reveals that R2R3MYB activation of flavonoid production in response to abiotic stress is an ancient character in land plants. New Phytol..

[43] Shen Y., Sun T., Pan Q., Anupol N., Chen H., Shi J., Liu F., Deqiang D., Wang C., Zhao J. (2019). RrMYB5- and RrMYB10-regulated flavonoid biosynthesis plays a pivotal role in feedback loop responding to wounding and oxidation in *Rosa rugosa*. Plant Biotechnol. J..

[44] Navrot N., Collin V.r., Gualberto J., Gelhaye E., Hirasawa M., Rey P., Knaff D.B., Issakidis E., Jacquot J.-P., Rouhier N. (2006). Plant glutathione peroxidases are functional peroxiredoxins distributed in several subcellular compartments and regulated during biotic and abiotic stresses. Plant Physiol..

[45] Li K., Xing C., Yao Z., Huang X. (2017). PbrMYB21, a novel MYB protein of *Pyrus betulaefolia*, functions in drought tolerance and modulates polyamine levels by regulating arginine decarboxylase gene. Plant Biotechnol. J..

[46] Ma D., Reichelt M., Yoshida K., Gershenzon J., Constabel C.P. (2018). Two R2R3-MYB proteins are broad repressors of flavonoid and phenylpropanoid metabolism in poplar. Plant J..

[47] Fan Z.-Q., Ba L.-J., Shan W., Xiao Y.-Y., Lu W.-J., Kuang J.-F., Chen J.-Y. (2018). A banana R2R3-MYB transcription factor MaMYB3 is involved in fruit ripening through modulation of starch degradation by repressing starch degradation-related genes and *MabHLH6*. Plant J..

[48] Zhu F., Luo T., Liu C., Wang Y., Yang H., Yang W., Zheng L., Xiao X., Zhang M., Xu R. (2017). An R2R3-MYB transcription factor represses the transformation of α- and β-branch carotenoids by negatively regulating expression of *CrBCH2* and *CrNCED5* in flavedo of *Citrus reticulate*. New Phytol..

[49] Lv Y., Yang M., Hu D., Yang Z., Ma S., Li X., Xiong L. (2017). The OsMYB30 transcription factor suppresses cold tolerance by interacting with a JAZ protein and suppressing β-amylase expression. Plant Physiol..

[50] Huang X.-S., Wang W., Zhang Q., Liu J.-H. (2013). A basic helix-loop-helix transcription factor, PtrbHLH, of *Poncirus trifoliata* confers cold tolerance and modulates peroxidase-mediated scavenging of hydrogen peroxide. Plant Physiol..

