# Supplementary material for: PtrMYB3, a R2R3-MYB Transcription Factor from Poncirus trifoliata, Negatively Regulates Salt Tolerance and Hydrogen Peroxide Scavenging
Source: Antioxidants (Basel). 2021 Aug 30;10(9):1388. doi: 10.3390/antiox10091388 (PMC8466168; doi:10.3390/antiox10091388)
Supplement: Supplementary file 1 [file antioxidants-10-01388-s001.zip › antioxidants-1358799-supplementary.pdf]

Table S1 Primer sequences used in this study

Primer names	Purpose	Forward primers (5'-3')	Reverse primers (5'-3')
PtrMYB3-T	Gene cloning	ATGGCGGGTAAGCGCAAGAC	TCACCAAAATCCCAAATCCG
PtrMYB3-pBI121	Overexpression	GAGAACACGGGGGACTCTAGAATGGCGGGTAAGCGCAAGAC	ATAAGGGACTGACCACCCGGGTCACCAAAATCCCAAATCCG
PtrMYB3-101LYFP	Subcellular localization	ATGGGATCTACTAGTGAATTCATGGCGGGTAAGCGCAAGAC	GGGGGTACCGTCGACGGATCCCCAAAATCCCAAATCCGATT
PtrMYB3-pGBKT7	Transactivation	ATGGCCATGGAGGCCGAATTCATGGCGGGTAAGCGCAAGAC	CCGCTGCAGGTCGACGGATCCCCAAAATCCCAAATCCGATT
PtrMYB3-pTRV2	VIGS	AGAAGGCCTCCATGGGGATCCAGAAGAAACCAAAGACCAG	TGTCTTCGGGACATGCCCGGGCCAAAATCCCAAATCCGATTGC
35S-F	Transgenic identification for tobacco	TCCTCGGATTCCATTGCCCAGC	
NPT II		CGGCTATGACTGGGCACAACA	CGGCAGGAGCAAGGTGAGATG
pTRV1	Positive identification for VIGS	ATTGAGGCGAAGTACGATGG	CCATCCACAATTATTTTCCGC
pTRV2-F		ATTCACTGGGAGATGATACGCT	
PtrMYB3-qPCR	qPCR	GAGGCACATTCCCAAAGCTG	TGTTCGTCCTGGAAGTCTCC
PtrPOD		ATCGCTCTTGCTGGAGACAG	TTCAAGGGGCATTCAGCCTC
PtrMYB3-62-SK	Dual-luciferase activity assay	CGCTCTAGAACTAGTGGATCCATGGCGGGTAAGCGCAAGAC	GATAAGCTTGATATCGAATTCTCACCAAAATCCCAAATCCG
pPOD-0800-Luc		GTCGACGGTATCGATAAGCTTGCACGTCATGATCCATGCCA	CGCTCTAGAACTAGTGGATCCCATTTTCTGATAAGCACTTGC
ACTIN	Internal reference	CCGACCGTATGAGCAAGGAAA	TTCCTGTGGACAATGGATGGA
Ubiquitin		GGTGTTTCCAGTGGCGGACG	TCCTCCCCTCAGCTACGGGGTAT

(Underlines indicate enzyme site.)

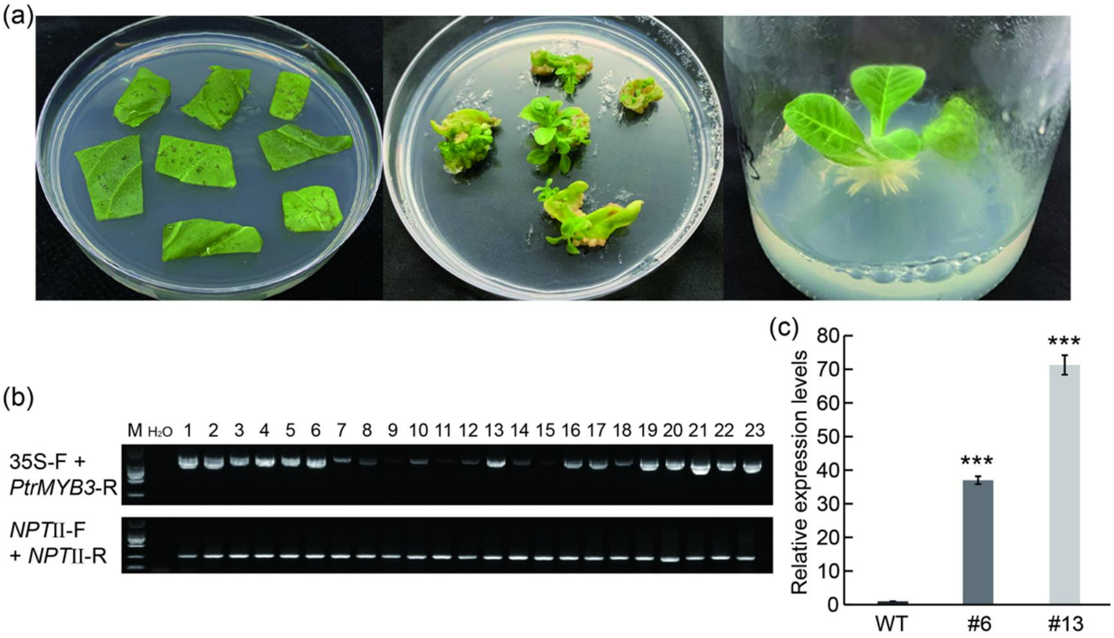

Figure S1. Genetic transformation and molecular identification of *PtrMYB3* overexpressed tobacco plants. (a) The representative pictures of genetic transformation. (b) The molecular identification of 23 transgenic tobacco plants using two pairs of primers. M: DNA marker. (c) The relative expression levels of *PtrMYB3* in WT and two transgenic lines (#6 and #13) measured by qPCR. Asterisks indicate significant differences between the transgenic line and WT: $p < 0.001$ (***).

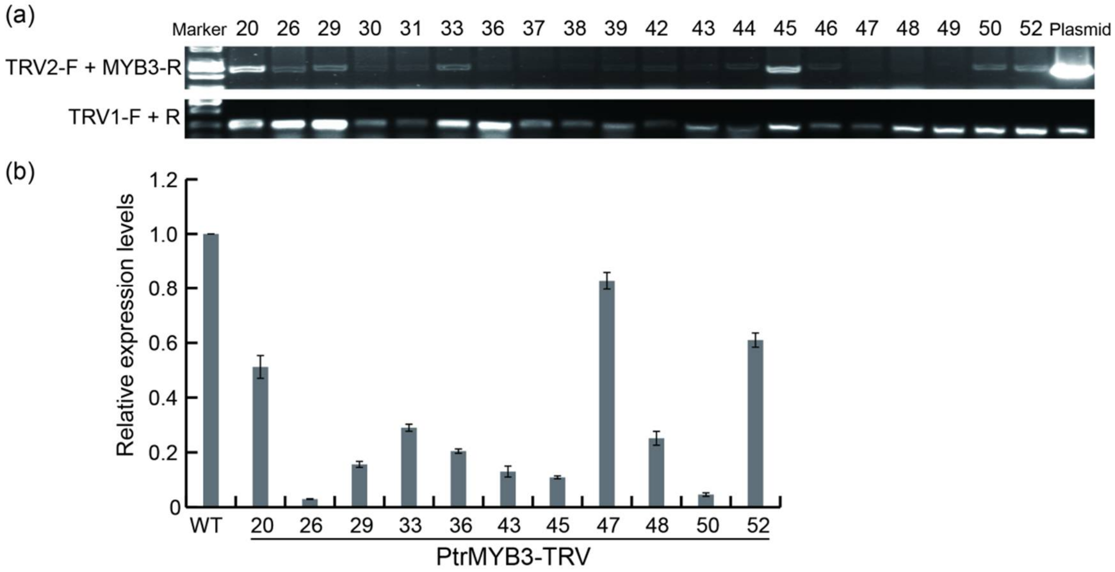

Figure S2. Molecular identification of *PtrMYB3*-silenced *Poncirus trifoliata* plants by genomic PCR and qPCR. (a) Genomic PCR of *PtrMYB3*-silenced plants using two pairs of primers. (b) The relative expression levels of WT and *PtrMYB3*-silenced plants (*PtrMYB3*-TRV) measured by qPCR.
